# Relation Between Immunohistochemical Expression of Hippo Pathway Effectors and Chronic Hepatitis Induced Fibrosis in Egyptian Patients

**DOI:** 10.5146/tjpath.2019.01463

**Published:** 2020-01-15

**Authors:** Rania Abdallah Abdallah, Mohammad Ibrahim Shaban, Doha Maher Taie, Nancy Youssef Asaad, Aya Hamdy Abd El Bary Badr

**Affiliations:** Department of Pathology, Menoufia University Faculty of Medicine, Shebein Elkom, Egypt; Department of Pathology, Menoufia University, Liver Institute, Menoufia, Egypt

**Keywords:** Yes-associated protein, Transcriptional coactivator with PDZ-binding motif, Chronic hepatitis, Fibrosis

## Abstract

***Objective:*** Chronic hepatitis is a global health problem especially in Egypt. Hepatic fibrosis is a common end clinical manifestation of many chronic liver diseases. Although it is a wound-healing process, excessive accumulation of fibrillary collagen leads to architectural damage, cirrhosis and liver failure. Recently, a few studies have linked Hippo pathway effectors of yes-associated protein (YAP) and its paralog transcriptional coactivator with PDZ-binding motif (TAZ) to extracellular matrix deposition and ongoing fibrosis.

***Material and Method:*** Immunohistochemical expression of YAP and TAZ were analyzed in 121 liver needle core biopsies (91 core biopsies of chronic viral hepatitis, 20 biopsies of autoimmune hepatitis and 10 normal liver cores).

***Results:*** YAP and TAZ nuclear localization was absent in all normal liver cores. Autoimmune hepatitis cases showed higher nuclear expression of both YAP and TAZ in comparison to chronic viral cases. YAP and TAZ expression were correlated with severity of hepatocyte injury together with fibrosis in chronic viral cases but these correlations were absent in AIH cases despite the pronounced increase of YAP and TAZ nuclear localization.

***Conclusion:*** The correlation between Hippo effectors activation and fibrosis in chronic viral hepatitis patients emphasize their role in the development and advancement of hepatic scarring and highlight the use of both YAP and TAZ as novel targets to ameliorate liver fibrosis.

## INTRODUCTION

Chronic hepatitis is an ongoing inflammation classified etiologically into chronic viral hepatitis, chronic non-viral hepatitis and diseases that mimic chronic hepatitis (1).

Chronic viral hepatitis is a major health problem worldwide (2), especially in Egypt (3). Hepatitis C virus (HCV) infection remains the most common cause of chronic hepatitis (4). Egypt has the highest prevalence of HCV infection in the world; 7.3% of the population has positive PCR (5). It represents a leading cause of liver-related deaths either due to end-stage liver disease or hepatocellular carcinoma (6). The global prevalence of hepatitis B virus (HBV) chronic carriers is about 3.6% of the general population (7). In Egypt, HBV prevalence was 1.4% of population [3]. Autoimmune hepatitis (AIH) incidence varies over time and place, according to risk factor prevalence (8). It is a relatively rare disease; about 1-2 per 100,000 populations per year are affected world-wide. Despite the availability of effective treatment, it remains a fatal disease with two-fold higher mortality than in the non-diseased (9).

Hepatic fibrosis is a common end clinical manifestation of many chronic liver diseases. Although it is a wound-healing process, excessive accumulation of fibrillary collagen leads to architectural damage, cirrhosis and liver failure (10). As a general rule, the available antifibrotic therapies (pegylated interferon (PEG-IFN) and IFN free therapy; Sofosbuvir) have been directed against suppressing hepatic inflammation rather than subduing fibrosis (11). No anti-liver fibrosis therapy has been approved so far (12,13).

Yes-associated protein (YAP) and its paralog transcriptional coactivator with PDZ-binding motif (TAZ) are key nuclear transcriptional effectors of Hippo pathway (14,15). The Hippo pathway was first discovered by genetic mosaic screens in Drosophila melanogaster (16,17) as an important regulator of cell growth, proliferation, differentiation, apoptosis and senescence (18,19). Components of this pathway are highly conserved in mammals (17). Under normal resting condition activated hippo kinase pathway sequestered inactive phosphorylated cytoplasmic YAP and TAZ, promoting their proteasomal degradation. Hippo pathway blockage released active YAP and TAZ with nuclear translocation and transcriptional activation by TEAD mediated complex (20,21). Their roles in liver regeneration (22) and carcinogenesis were well established (23). Inactivation of Hippo signaling and enhanced YAP expression and activity are critical for liver regenerative capacity and this occurred only in stress conditions (22,24). A few recent studies have described YAP and TAZ as mechanosensitive coordinators of the matrix-driven feedback loop that initiates and sustains fibrosis (25).

Therefore, this study aimed to assess the immunohisto-chemical expression of YAP and TAZ in chronic viral hepatitis together with AIH patients and verify their roles in the development of hepatic fibrosis.

## MATERIAL and METHODS

This retrospective study included 121 needle liver biopsies of Egyptian patients, obtained from the archival material of Pathology Department, National Liver Institute, Menoufia University. The studied cases were classified into three groups as follows: The control group included 10 normal biopsies obtained from donors for liver transplantation. The chronic viral hepatitis group included 91 biopsies divided into 54 specimens from patients positive for HCV but negative for HBV infection and 37 specimens from patients positive for HBV and negative for HCV infection proved by ELISA. The AIH group included 20 biopsies from patients negative for HCV and HBV infection by serological tests but proved to be AIH based on laboratory data. Totally all cases had a >10 score according to the Revised International Autoimmune Hepatitis Group modified scoring system (26). All cases were negative for bilharziasis by serologic tests.

Demographic, radiological and laboratory data were collected from patients’ medical records and included age (years), gender, data of ultrasound (US) examination of liver and spleen, ALT, AST, ALP, serum total bilirubin, total albumin and serum HCV RNA level by quantitative PCR.

### Histopathological Analysis

The Ishak scoring system was adopted for evaluation of necro-inflammatory changes (grades) and architectural changes (stages) (27).

1. Portal tract changes: The degree of portal inflammation was divided into scores of 0, 1,2,3,4 and then grouped for statistical purposes as mild/moderate inflammation with score (1,2) and moderate/marked inflammation with score (3,4). Lymphoid aggregate and bile duct injury was reported as present or absent. Plasma cell infiltrate was grouped as ≥50% and <50% of total portal tracts infiltrate (28). The presence of ductular proliferation was evaluated as focal or diffuse.
2. Parenchymal changes: Interface hepatitis was grouped for statistical purposes as mild/moderate focal; score (1,2) and moderate/severe continuous; score (3,4). The degree of spotty necrosis was grouped into score (0), score (1,2) and score (3 and 4). Confluent necrosis was also grouped as score (0), scores (1,2), scores (3,4) but score (5 and 6) was omitted as there was no specimen had this score. Steatosis was graded according to the Brunt’s grading system, based on the percentage of involved hepatocytes in the biopsy specimen as follows: Grade 0 = none, grade 1 (mild) = up to 33%, grade 2 (moderate) = up to 66% and grade 3 (severe) = more than 66% (29). These grades were lumped into three categories for statistical purposes as follows: grade (0), grade (1,2) and grade (3). Emperipolesis was reported as present or absent. Rosettes formation was classified as involving ≥50% and <50% of total parenchymal cells (28).
3. Grades of necro-inflammatory changes (HAI grading) were grouped into two categories for statistical purposes: minimal/mild activity; score (1-8) and moderate/severe activity; score (9-18).
4. Stages of architectural changes (fibrosis score) were also grouped for statistical purposes as follows: no fibrosis: score (0), portal and periportal fibrosis: score (1,2), bridging fibrosis: score (3,4) and severe fibrosis and cirrhosis: score (5,6).

### Immunohistochemistry

Several paraffin sections, each one 4 um in thickness, were cut from each case, one section for hematoxylin and eosin staining and the others for immunohistochemical process. The method used for immunostaining was streptavidin–biotin amplified system. Paraffin-embedded tissue sections were deparaffinized in xylene, rehydrated in a graded series of ethanol, and then incubated with 3% hydrogen peroxide. Slides were rinsed in phosphate-buffered saline (PBS) and then exposed to heat-induced epitope retrieval in citrate buffer solution (pH 6) for 20 minutes. After cooling, the slides were incubated overnight at room temperature with rabbit polyclonal anti-YAP antibody (Cat.N. sc-15407, Santa Cruz, INC) (1 ml concentrated and diluted by PBS in a dilution 1:75) and rabbit polyclonal TAZ (Cat.N. sc-48805, Santa Cruz, INC) (1 ml concentrated and diluted by PBS in a dilution 1:75). Positive tissue controls were normal human kidney for YAP and normal human gall bladder for TAZ. Detection of immunoreactivity was carried out using the Universal Dakocytomation Labelled streptavidin–Biotin-2 system, horseradish Peroxidase (LSAB-2 System, HRP Kit, Catalogue No. k0679). Finally, the reaction was visualized by an appropriate substrate/chromogen (diaminobenzidine) reagent. Counter stain was carried out using Mayer’s hematoxylin. The staining procedure included negative controls obtained by substitution of primary antibodies with phosphate-buffered saline.

### Interpretation of Immunostaining Results

The positively stained cells were characterized by presence of brownish nuclear coloration detected by DAB reaction (19) and the expression was assessed in liver hepatocytes where any number of positive cells was required to assign their positivity (30). The H score system was applied according to (31), where both the intensity and percentage of positivity were considered using the following formula: H score = (3×% of strong intensity) + (2 ×% of moderate intensity) + (1 ×% of mild intensity)

The intensity of expression was ranked as: 0, no staining; +1, mild intensity; +2, moderate intensity; and +3, strong intensity.

### Statistical Analysis

Data were collected, tabulated and statistically analyzed using a personal computer with SPSS “Statistical Package for the Social Sciences” program for Windows, version 18, SPSS Inc., Chicago, Illinois, USA. A p value ≤0.05 was considered statistically significant.

## RESULTS

Demographic, radiological and laboratory findings of the studied both chronic viral and autoimmune hepatitis groups were presented in Table 1 while histopathological data of both groups were presented in Table 2.

**Table 1:** Demographic, radiological and laboratory findings of the studied both chronic viral and autoimmune hepatitis groups.

**Variables**	**Chronic viral hepatitis**	**Autoimmune hepatitis**
	**n (%)**	**n (%)**
	**91 (100)**	**20 (100)**
**Age (years)**		
Range	13-57	1‐47
Median	34	11
Mean ± SD	35.2±10.9	15±13.9
**Gender**		
Male	68 (74.7)	9 (45)
Female	23 (25.3)	11 (55)
Male/female	2.9/1	1/1.2
**Liver US**		
Average	53 (58.2)	4 (20)
Hepatomegaly	27 (29.7)	11 (55)
Cirrhotic	11 (12.1)	5 (25)
**Spleen US**		
Average	77 (84.6)	10 (50)
Splenomegaly	14 (15.4)	10 (50)
**ALT (U/L)**		
Range	13-259	24-933
Median	43	77
Mean ± SD	50.9±35.8	207.8±270
**AST (U/L)**		
Range	11-173	33-900
Median	37	82
Mean ± SD	42.9±28	198.7±236.5
**ALP (U/L)**		
Range	40-180	44-250
Median	47	82
Mean ± SD	56.9±23.9	121.5±83.2
**Total bilirubin (mg/dl)**		
Range	0.1-3	0.4-17
Median	0.7	1.7
Mean ± SD	0.8±0.4	4.4±5.4
**Albumin (gm/dl)**		
Range	2-3.8	1.8-3.5
Median	3	2.3
Mean ± SD	3.1±0.4	2.5±0.5
**PCR**		
Range	4000-64300000	
Median	543500	
Mean ± SD	3606411.4±9651163.9	

**US:** Ultrasound, **PCR:** Polymerase chain reaction, **ALT:** Alanine transaminase, **AST:** Aspartate transaminase, **ALP:** Alkaline phosphatase

**Table 2:** Histopathological data of the studied both chronic viral and autoimmune hepatitis groups.

**Variables**		**Chronic viral hepatitis**	**Autoimmune hepatitis**
		**n (%)**	**n (%)**
		**91 (100)**	**20 (100)**
**Portal features**	**Portal inflammation**		
	Score (1,2)	73 (80.2)	11 (55)
	Score (3,4)	18 (19.8)	9 (45)
	**Lymphoid aggregate**		
	Absent	56 (61.5)	15 (75)
	Present	35 (38.5)	5 (25)
	**Plasma cell infiltrate**		
	<50%	73 (80.2)	10 (50)
	≥50%	18 (19.8)	10 (50)
	**Bile duct injury**		
	Absent	46 (50.5)	6 (30)
	Present	45 (49.5)	14 (70)
	**Ductular proliferation **		
	Focal	74 (94.9)	4 (20)
	Diffuse	17 (5.1)	16 (80)
**Parenchymal features**	**Interface hepatitis **		
	Score (1,2)	88 (96.7)	11 (55)
	Score (3,4)	3 (3.3)	9 (45)
	**Spotty necrosis**		
	Score (0)	4 (4.4)	1 (5)
	Score (1,2)	87 (95.6)	16 (80)
	Score (3,4)	0 (0)	3 (15)
**Parenchymal features**	**Confluent necrosis**		
	Score (0)	44 (48.4)	7 (35)
	Score (1,2)	46 (50.5)	12 (60)
	Score (3,4)	1 (1.1)	1 (5)
	**Steatosis**		
	Grade (0)	52 (57.1)	19 (95)
	Grade (1,2)	35 (38.5)	1 (5)
	Grade (3)	4 (4.4)	0 (0)
	**Emperipolesis**		
	Absent	75 (82.4)	8 (40)
	Present	16 (17.6)	12 (60)
	**Rosettes**		
	<50%	74 (81.3)	11 (55)
	≥50%	17 (18.7)	9 (45)
**Grade of necroinflammation**			
Score (1-8)		82 (90.1)	11 (55)
Score (9-18)		9 (9.9)	9 (45)
**Stage of fibrosis**			
Score (0)		5 (5.5)	0 (0)
Score (1,2)		56 (61.5)	4 (20)
Score (3,4)		16 (17.6)	11 (55)
Score (5,6)		14 (15.4)	5 (25)

### Immunohistochemical Expression of YAP and TAZ in Control, Chronic and Autoimmune Hepatitis Groups

**Figure 1:**
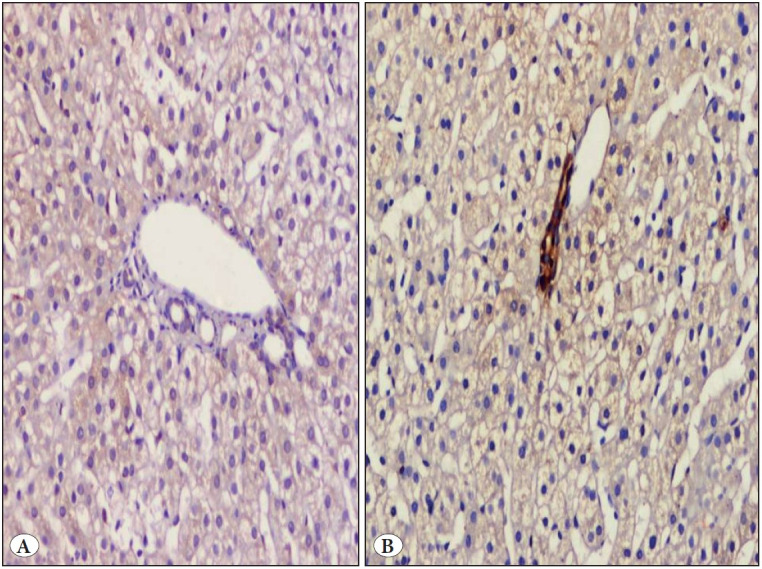
A healthy subject (donor for liver transplantation) showed negative nuclear expression for **A)** YAP and **B)** TAZ within hepatocytes (IHC; x200).

*I. Control group: *None of the studied control normal liver tissues showed nuclear staining of either YAP or TAZ within the hepatocytes (0/10 cases) (Figure 1).

*II. Chronic viral hepatitis group: *Sixty three cases out of 91 (69.2%) showed positive nuclear staining of hepatocytes for active YAP while eighty two cases (82/91) (90.1%) showed positive nuclear staining for active TAZ. The nuclei of bile ducts showed positive expression in (3/91) (3.3%) for YAP and in 33/91 cases (36.3%) for TAZ. Moreover, brown nuclear staining of portal tract fibroblasts was observed in 2/91 (2.2%) for YAP and in 38/91 cases (41.8%) for TAZ.

*III. Autoimmune hepatitis group (AIH): *Nuclear YAP positivity was observed in hepatocytes of only 15/20 AIH cases (75%), while all specimens of AIH group (100%) showed TAZ positivity within hepatocytes.

All the studied AIH cases showed absence of nuclear YAP positivity in either portal bile duct epithelium or fibroblasts. On the other hand, TAZ nuclear staining appeared in 12/20 cases (60%) of portal bile ducts and in 14/20 cases (70%) within portal fibroblasts.

### Comparison Between the Control, Chronic Viral and Autoimmune Hepatitis Groups Regarding the YAP and TAZ Expression

**Figure 2:**
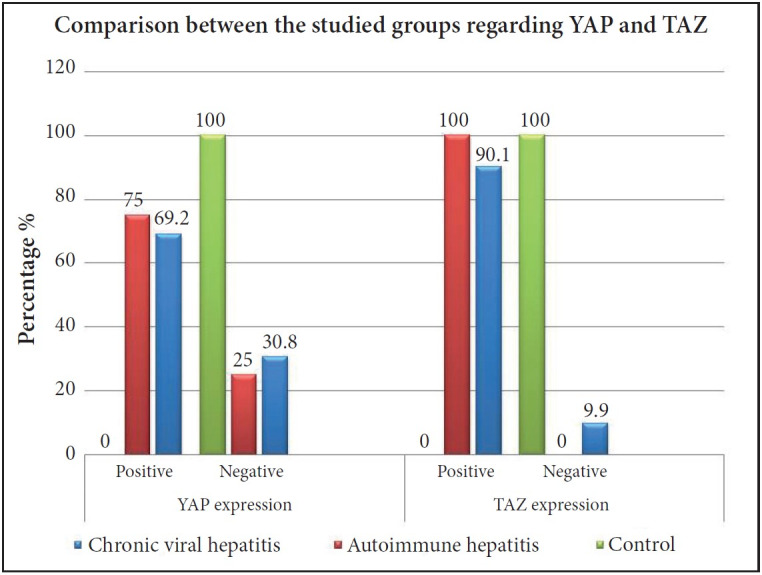
Comparison between the studied groups regarding YAP and TAZ expression (p<0.001).

**Figure 3:**
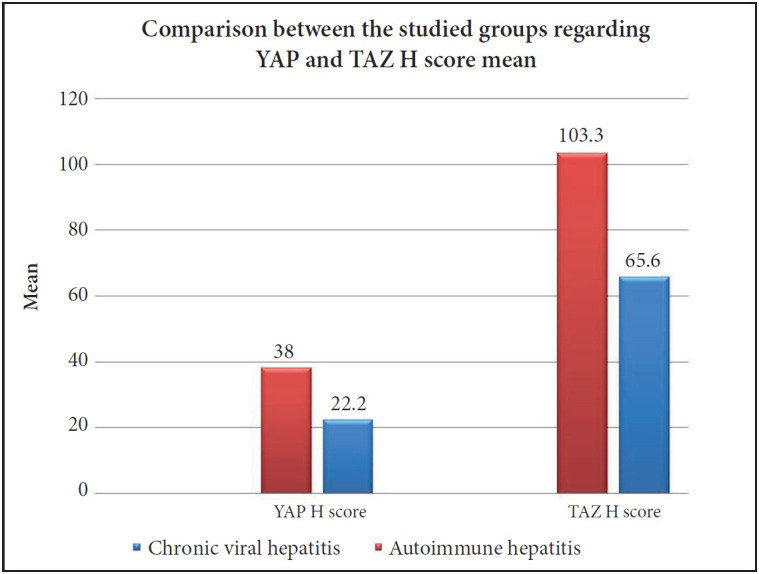
Comparison between the studied groups regarding YAP and TAZ H score mean (p=0.04 and p=0.004 respectively).

There was a high statistically significant difference between the studied groups regarding both nuclear YAP and TAZ positivity. All the studied control cases showed negative expression of both YAP and TAZ in comparison to 69.2% and 90.1% of chronic viral hepatitis group cases positive for YAP and TAZ respectively and the percent increased to reach 75% and 100% (respectively) in the AIH group (p= <0.001), (Figure 2). Also, the AIH group exhibited higher H score values of both YAP and TAZ in comparison to the chronic viral hepatitis group (p=0.04 and p=0.004 respectively), (Figure 3).

### Association Between YAP and TAZ H Scores with the Studied Clinicopathological Parameters in the Chronic Viral Hepatitis Group

*Clinically*, only the liver US findings displayed a significant association with both YAP and TAZ H score values as progressive increase in mean values of H scores were noticed with advancement of liver pathology (p= 0.006, p=0.003 respectively) (Table 3).

**Table 3:** The relationship of YAP and TAZ H scores in chronic viral hepatitis group and the studied clinicopathological parameters.

		**YAP H score in chronic viral hepatitis**			**TAZ H score in chronic viral hepatitis**		
**Variables**		**Mean±SD**	**Test of significance**	**p value**	**Mean±SD**	**Test of significance**	**p value**
**Gender**			Independent samples t-test=0.3	0.6		Independent sample t-test=0.3	0.4
Male		21.1±28.7			62.8±53.3		
Female		25.2±32.4			73.9±49.2		
**Liver US**			One way anova=5.4	0.006**HS		One way anova=6.2	0.003**HS
Average		14.7±21.8		#P1=0.04*S	51.5±43.3		#P1=0.03*S
Hepatomegaly		28.5±37		P2=0.003**HS	77.8±60.8		P2=0.002**HS
Cirrhotic		42.7±30.8		P3=0.2	103.6±46.5		P3=0.1
**Spleen US**			Independent samples t-test=1.1	0.2		Independent sample t-test=1	0.5
Average		20.3±27.9			67.2±53.7		
Splenomegaly		32.5±36.5			56.8±44.2		
**Portal features**	**Portal inflammation **		Independent samples t-test=7.7	0.04*S		Independent sample t-test=0.6	0.5
	Score (1,2)	19±25.5			63.6±51.5		
	Score (3,4)	35±40.6			73.6±56.1		
	**Lymphoid aggregate **		Independent samples t-test=24	0.004**HS		Independent sample t-test=1	0.05*S
	Absent	14±18.1			57.1±50		
	Present	35.3±38.6			79.1±53.6		
	**Bile duct injury**		Independent samples t-test=4.7	0.3		Independent sample t-test=0.3	0.09
	Absent	25.1±33.8			74.7±53.9		
	Present	19.2±24.5			56.3±49.4		
	**Ductular proliferation**		Independent samples t-test=11.6	0.03*S		Independent sample t-test=2.4	0.03*S
	Focal	17.6±23.8			59.9±49.4		
	Diffuse	42.1±42.5			90.3±58.7		
**Parenchymal features**	**Interface hepatitis **		Independent samples t-test=0.3	0.2		Independent sample t-test=0.4	0.9
	Score (1,2)	21.5±29.6			65.8±52.7		
	Score (3,4)	43.3±20.8			60±45.8		
	**Spotty necrosis**		One way anova=0.7	0.4		One way anova=1.3	0.3
	Score (0)	10±13.5			36.3±32.5		
	Score (1,2)	22.7±29.9			66.9±52.7		
	Score (3,4)	0			0		
	**Confluent necrosis**		One way anova=8.5	< 0.001**HS		One way anova=6.01	0.004**HS
	Score (0)	10±12.5			48.1±44.2		
	Score (1,2)	33.5±36.2			80.8±54.2		
	Score (3,4)	40±-			140±-		
	**Steatosis**		One way anova=4.4	0.02*S		One way anova=3.3	0.04*S
	Grade (0)	14.8±23.1		##P1=0.004**HS	54.9±44.4		##P1=0.01**HS
	Grade (1,2)	33.3±35.5		P2=0.7	83±60.8		P2=0.9
	Grade (3)	21.3±21.8		P3=0.4	52.5±28.7		P3=0.3
	**Rosettes**		Independent samples t-test=5.5	0.2		Independent sample t-test=2.7	0.1
	<50%	20.1±26.5			63.7±49.7		
	≥50%	31.5±39.7			73.8±63.3		
**Grade of necroinflammation**			Independent samples t-test=1.2	0.04*S		Independent sample t-test=0.4	0.7
Score (1-8)		20.4±27.8			64.9±53		
Score (9-18)		38.9±40.4			72.2±47.1		
**Stage of fibrosis**			One way anova=12.7	<0.001**HS		One way anova=4.495	0.006**HS
Score (0)		4±4.2		###P1=0.5	72±64.2		###P1=0.4
Score (1,2)		11.5±15.1		P2=0.002*HS	50.98±44.7		P2=0.4
Score (3,4)		45.3±38.1		P3=0.002**HS	94.1±56.7		P3=0.5
				P4<0.001**HS			P4=0.003**HS
				P5=0.001**HS			P5=0.01**HS
Score (5,6)		45±39.3		P6=0.9	89.3±54.1		P6=0.8

**SD:** Standard deviation, **US:** ultrasound, **HS:** Highly significant. # Post-hoc test: P1: difference between average and hepatomegaly, P2: difference between average and cirrhotic, P3: difference between hepatomegaly and cirrhotic, S: Significant. ## Post-hoc test: P1: difference between score (0) and score (1,2), P2: difference between score (0) and score (3), P3: difference between score (1,2) and score (3). ### Post-hoc test: P1: difference between score (0) and score (1,2), P2: difference between score (0) and score (3,4), P3: difference between score (0) and score (5,6), P4: difference between score (1,2) and score (3,4), P5: difference between score (1,2) and score (5,6), P6: difference between score (3,4) and score (5,6).

**Table 4:** Pearson’s correlation of YAP and TAZ H scores in chronic viral, autoimmune hepatitis groups and the laboratory findings.

**Variables**	**YAP H score in chronic viral hepatitis**		**TAZ H score in chronic viral hepatitis**		**YAP H score (autoimmune hepatitis)**		**TAZ H score (autoimmune hepatitis)**	
	**r**	**p value**	**r**	**p value**	**r**	**p value**	**r**	**p value**
Age (years)	0.3	0.001**HS	0.1	0.3	0.2	0.3	0.1	0.6
ALT (U/L)	0.4	<0.001**HS	0.3	0.005**HS	0.7	0.002**HS	0.3	0.2
AST (U/L)	0.5	<0.001**HS	0.3	0.008**HS	0.7	<0.001**HS	0.2	0.3
ALP (U/L)	0.4	<0.001**HS	0.2	0.03*S	0.6	0.002**HS	0.4	0.06
Bilirubin (mg/dl)	0.07	0.5	0.04	0.7	0.1	0.7	0.1	0.6
Albumin (gm/dl)	-0.3	0.006**HS	0.1	0.2	0.2	0.4	0.3	0.2

* r: Pearson’s correlation, **ALT:** alanine transaminase, **AST:** aspartate transaminase, **ALP:** alkaline phosphatase, **HS: highly significant, *S: significant.

*Regarding portal changes*, higher scores (3, 4) of portal tract inflammation was associated with increased YAP H score only (p=0.04). Also higher both YAP and TAZ H score values tended to show a significant association with lymphoid aggregate formation (p= 0.004 and p=0.05 respectively) together with the appearance of diffuse ductular proliferation (p=0.03) (Table 3).

**Figure 4:**
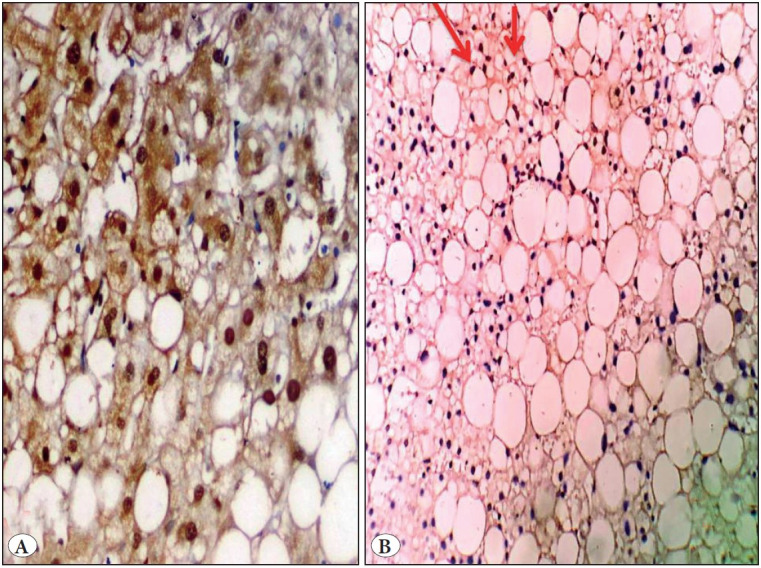
Chronic viral hepatitis. **A)** Mild steatosis of hepatic parenchyma; grade (1) showed diffuse strong nuclear expression for TAZ within hepatocytes (IHC;x400). **B)** Marked steatosis; grade (3) showed scattered nuclear expression for YAP (red arrows) (IHC; x200).

*Regarding parenchymal changes*, both YAP and TAZ H score values were significantly correlated with cases displaying higher scores of confluent necrosis (p<0.001 ) and mild/moderate grades of steatosis (grade 1, 2) compared to a severe grade of steatosis (grade 3) (Figure 4), (p=0.01 and p=0.02 respectively) (Table 3).

**Figure 5:**
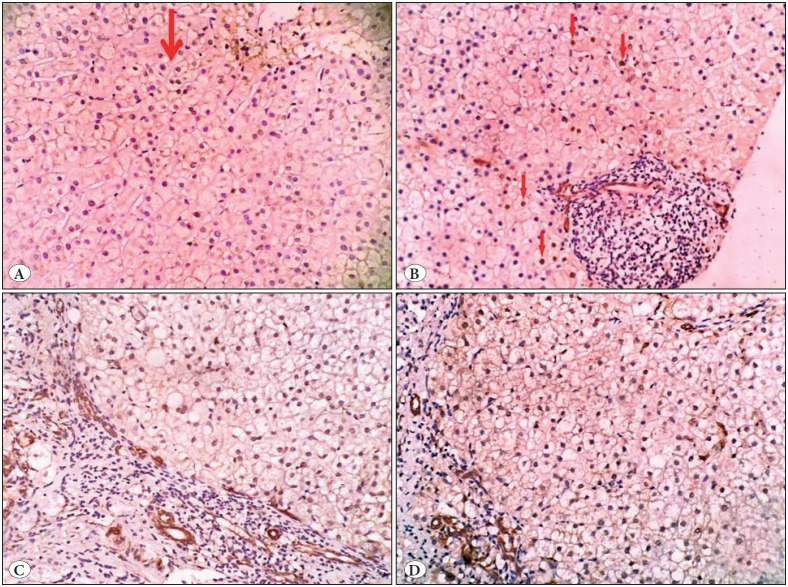
Scattered mild nuclear expression within hepatocytes (red arrows) **A)** for YAP and **B)** for TAZ in chronic viral hepatitis showing mild inflammatory activity (5/18) and mild fibrosis (1/6). Diffuse strong nuclear expression **C)** for YAP and **D)** for TAZ in chronic viral hepatitis with marked inflammatory activity (14/16) and marked fibrosis (6/6); cirrhosis. (IHC; x200).

The mean H score value of YAP showed significant progressive elevation with increasing grade of necro-inflammation (p=0.04), (Figure 5, Table 3).

Higher stages of fibrosis of chronic viral hepatitis cases were also statistically associated with higher means of both YAP and TAZ H-score values (p <0.001) (Table 3, Figure 5).

A significant positive correlation between YAP H score and age was observed (p=0.001). Moreover, both YAP and TAZ H score values exhibited also positive correlation with serum level of ALT (p<0.001 and p=0.005 respectively), AST (p<0.001 and p<0.008 respectively) and ALP (p<0.001 and p<0.03 respectively). This correlation became negative regarding albumin serum level and YAP H score value (p=0.006), (Table 4).

Linear regression revealed that AST level and fibrosis stages were the most independent parameters predicting YAP H score values in the chronic viral hepatitis group (p=0.009 and p= 0.008 respectively). They cause changes in YAP H score values by 30%. In the same line, TAZ H score linear regression analysis revealed that liver US, confluent necrosis and fibrosis stages were the most independent factors in predicting TAZ H score values in the chronic viral hepatitis group (p=0.02, p=0.02 and p=0.04 respectively). They share in TAZ H score value changes by 20%, (Table 5).

**Table 5:** Linear regression of YAP and TAZ H scores in chronic viral hepatitis group and the significant variables.

**Variables**	**YAP H score in chronic viral hepatitis**				**TAZ H score in chronic viral hepatitis**			
	**B**	**CI**	**SE**	**p value**	**B**	**CI**	**SE**	**p value**
Age (years)	0.4	-0.2-1	0.3	0.1				
ALT (U/L)	-0.3	-0.6-0.03	0.2	0.07	0.2	-0.1-0.5	0.1	0.2
AST (U/L)	0.6	0.1-1	0.2	0.009**HS	0.14	-0.7-0.8	0.4	0.9
ALP (U/L)	0.2	-0.2-0.5	0.2	0.4	-0.2	-0.8-0.5	0.3	0.6
Albumin (gm/dl)	-7.6	-21.2-5.9	6.8	0.3				
Liver US	-4.1	-14.4-6.2	5.2	0.4	19	3.4-34.6	7.8	0.02*S
Portal inflammation	-17.8	-36.5-0.8	9.4	0.06				
Lymphoid aggregate	-1.3	-16.3-13.6	7.5	0.9	-2.5	-28.8-23.6	13.2	0.8
Ductular proliferation	-9.2	-29.3-10.9	10.1	0.4	-5.9	-40.5-28.5	17.3	0.7
Confluent necrosis	11.9	-0.4-24.2	6.2	0.06	24.2	3.2-45	10.5	0.02*S
Steatosis	4	-6.2-14.2	5.1	0.4	6.2	-11.6-24.2	9	0.5
Grades of necroinflammation	-18.6	-43.5-6.3	12.5	0.1				
Stages of fibrosis	17.3	4.7-29.9	6.3	0.008**HS	0.4	-22.8-23.6	11.6	0.04*S

*YAP H score= B0 + (B1 x age) + (B2 x ALT) + (B3 x AST) +…………… *Adjusted R^2^=0.3 * B0= 39.3 *TAZ H score= B0 + (B1 x ALT) + (B2 x AST) + (B3 x ALP) +…………… *Adjusted R^2^=0.2 * B0= 23.6 *B: Regression coefficient, **CI:** Confidence interval for B, **SE:** Standard error, **ALT:** Alanine transaminase, **AST:** aspartate transaminase, **ALP:** alkaline phosphatase, **US:** ultrasound, **R^2^:** correlation factor^2^, **B0:** B constant, ** **HS:** Highly significant, *S: Significant.

### Correlation Between YAP and TAZ Immunostaining Results of the Studied Chronic Viral Hepatitis Group

A highly statistical significant strong direct correlation was observed between YAP and TAZ H score values in chronic viral hepatitis cases (p <0.001) where increased values of the YAP H score were associated with increased values of TAZ (Table 6).

**Table 6:** Pearson’s correlation between YAP and TAZ H scores of parenchymal expression in the studied chronic viral and autoimmune hepatitis groups.

**Chronic viral hepatitis**		**YAP H score**
**TAZ H score**	**r**	0.5
	**p value**	<0.001**HS
**Autoimmune hepatitis**		**YAP H score**
**TAZ H score**	**r**	0.3
	**p value**	0.2

**r:** Pearson correlation, **HS: Highly significant

### Relationship Between YAP and TAZ H Scores and the Studied Clinicopathological Parameters in AIH

**Figure 6:**
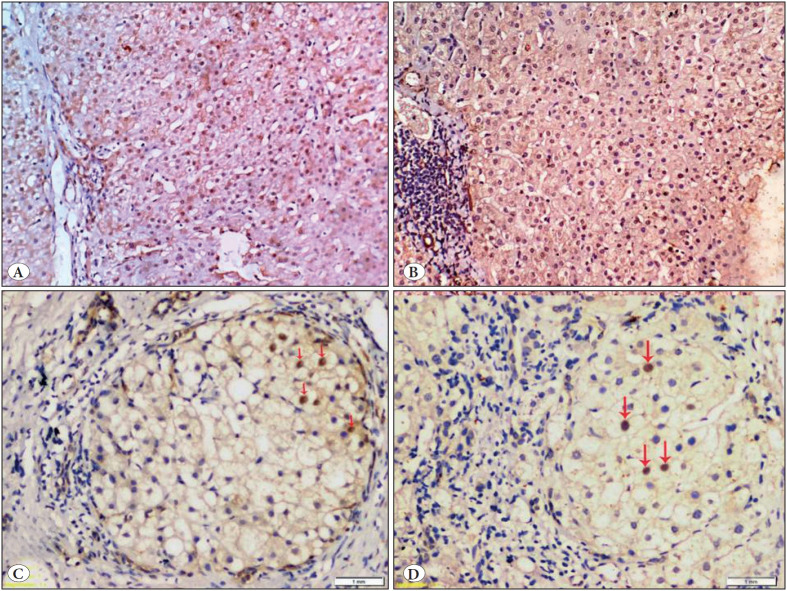
Diffuse strong nuclear expression within hepatocytes **A)** for YAP and **B)** for TAZ in autoimmune hepatitis (AIH) with moderate inflammatory activity (8/18) and mild fibrosis (2/6). Scattered mild nuclear expression **C)** for YAP and **D)** for TAZ in AIH with mild inflammatory activity (6/18) and cirrhosis (6/6) (red arrows), (IHC; x200).

Both YAP and TAZ H score values failed to show any statistical significant association with the studied parameters, Table 7 (Figure 6).

**Table 7:** The relationship of YAP and TAZ H scores in autoimmune hepatitis group and the studied clinicopathological parameters.

**YAP H score in autoimmune hepatitis group**					**TAZ H score in autoimmune hepatitis group**		
**Variables**		**Mean ± SD**	**Test of significance**	**p value**	**Mean ± SD**	**Test of significance**	**p value**
**Gender**			Independent sample t-test=0.09	0.5		Independent sample t-test=0.05	0.3
Male		32.2±34.2			90±44.7		
Female		42.7±42.9			114.1±49.9		
**Liver US**			One way anova=1.9	0.2		One way anova=0.3	0.8
Average		70±24.5			87.5±61.8		
Hepatomegaly		28.2±26.4			105±50.4		
Cirrhotic		34±59.8			112±37		
**Spleen US**			Independent sample t-test=0.5	1		Independent sample t-test=0.2	0.5
Average		38±33.3			111.5±50.8		
Splenomegaly		38±45.2			95±46.2		
Portal features	**Portal inflammation**		Independent sample t-test=0.4	0.8		Independent sample t-test=2.1	0.3
	Score (1,2)	36.4±34.4			92.7±52.4		
	Score (3,4)	40±45.3			116.1±41.4		
	**Lymphoid aggregate**		Independent sample t-test=2.7	0.3		Independent sample t-test=0.4	0.2
	Absent	42.7±42.7			95.7±46.6		
	Present	24±19.5			126±49.8		
	**Bile duct injury**		Independent sample t-test=5.9	0.3		Independent sample t-test=0.6	0.6
	Absent	28.3±17.2			111.7±56.7		
	Present	42.1±44.8			99.6±45.7		
	**Ductular proliferation**		Independent sample t-test=0.1	0.6		Independent sample t-test=8	0.9
	Focal	47.5±35.9			107.5±72.7		
	Diffuse	35.6±39.9			102.2±43.1		
Parenchymal features	**Interface hepatitis**		Independent sample t-test=1.9	0.1		Independent sample t-test=0.3	0.9
	Score (1,2)	41.8±34.6			104.1±49.4		
	Score (3,4)	20±21.4			100±52.1		
	**Spotty necrosis**		One way anova=0.2	0.8		One way anova=1.2	0.3
	Score (0)	20±0			50±0		
	Score (1,2)	36.8±41.7			110.9±46.7		
	Score (3,4)	50±26.4			80±52.9		
	**Confluent necrosis**		One way anova=0.9	0.4		One way anova=0.7	0.5
	Score (0)	44.3±37.8			117.1±50.9		
	Score (1,2)	30.8±39.4			98.8±47.5		
	Score (3,4)	80±-			60±-		
	**Steatosis**		One way anova=0.5	0.5		One way anova=0.6	0.4
	Grade (0)	39.5±39.1			101.3±48.5		
	Grade(1,2)	10±-			140±-		
	Grade (3,4)	0			0		
	**Rosettes**		Independent sample t-test=0.03	0.6		Independent sample t-test=0.3	0.8
	<50%	41.8±36.3			100.5±51.9		
	≥50%	33.3±43			106.7±45.5		
**Grade of necroinflammation**			Independent sample t-test=0.3	0.8		Independent sample t-test=2.1	0.3
Score (1-8)		36.4±34.4			92.7±52.4		
Score (9-18)		40±45.3			116.1±41.4		
**Stage of fibrosis**			One way anova=0.4	0.7		One way anova=0.6	0.5
Score (0)		0			0		
Score (1,2)		52.5±34			80±66.8		
Score (3,4)		32.7±29.7			112.3±42.3		
Score (5,6)		38±60.9			102±48.7		

**SD:** Standard deviation, **US:** Ultrasound

A positive and strong highly significant correlation was found between serum levels of ALT, AST and ALP and YAP H score (p=0.002, p<0.001 and p=0.002 respectively) (Table 4).

### Correlation Between YAP and TAZ Immunostaining Results of the Studied AIH group

No correlation was observed between YAP H score and TAZ H score values in the studied AIH cases (Table 6).

## DISCUSSION

In our study, donor control liver tissues showed neither YAP nor TAZ nuclear staining within hepatocytes and this was similar to several studies that showed absence of YAP nuclear staining in healthy mature hepatocytes (32,33). Moreover, YAP and TAZ have been reported to be prominently expressed in fibrotic but not healthy lung tissue (25). This could occur because the hippo pathway was activated during the resting condition, causing suppression of YAP and TAZ nuclear translocation (20,21).

In the studied chronic viral hepatitis cases, YAP and TAZ were expressed in hepatocytes which may occur in response to their injury by the virus (33). YAP and TAZ were also expressed mostly in the cytoplasm together with the nuclei of both portal fibroblasts and bile ducts. This recapitulated the study of Mannaerts, et al. (19). Also Machado, et al. (33) showed that YAP and TAZ were expressed in the cytoplasm and nuclei of bile ducts. Adding to that , although YAP appeared overexpressed in fibroblasts of the Dupuytren fibrotic diseased cases, this overexpression did not correlate with the number of activated fibroblasts that were positive to αSMA )smooth muscle actin) which lay down collagen I responsible for fibrosis (34). Thus the difference in the percentage of YAP and TAZ nuclear positivity between hepatocyte and portal fibroblasts in our cases may be related to the chronic nature of viral hepatitis together with early action of YAP in activating pro-fibrotic genes in cultured activated HSCs reaching the strongest induction 10 hours after activation and then showing an expression decline but with the target genes continuing their overexpression (19).

In AIH group; both YAP and TAZ nuclear localization were seen in hepatocytes, however, portal fibroblasts and bile ducts showed nuclear staining only for TAZ. This could be explained by the superior inflammatory role of TAZ in the autoimmune process (35). Furthermore, fibroblasts are responsible mainly for initiation of the earliest molecular events leading to inflammatory autoimmune responses secreting type 1 insulin-like growth factor receptor (IGF-1R) that acts as chemoattractant to T cells (36).

In the process of autoimmunity, hippo pathway effectors appeared to play a key role. Enger et al. (37) found that Sjogren’s autoimmune salivary gland disease occurred mainly due to loss of salivary cell junctional integrity via dysregulation of Hippo signaling pathway. Normal cyto-differentiation and organization of acinar and ductal salivary gland structures depends on interaction of cytoplasmic YAP and TAZ with E-cadherin and α-catenin which are important regulators of cellular polarity. So, in case of a defect in the Hippo pathway, YAP and TAZ activation plays a pivotal role in the development of this disease.

Moreover, a recent study demonstrated a novel role of TAZ in autoimmunity through inducing imbalance between regulatory T cells (T reg) and inflammatory TH17. TAZ alone and not YAP causes stimulation of TH17 proliferation and promote T reg degradation (35). TAZ performs this function through retinoid-related orphan receptor gamma (RORγt) and forkhead box P3 (Foxp3) transcription factors (38). This important role of hippo effectors in autoimmunity was reflected in the studied AIH cases that showed higher YAP and TAZ expression in addition to higher H score values in comparison to chronic viral hepatitis cases.

In the chronic viral hepatitis group, radiological ultrasound findings showed progressive increase in both YAP and TAZ H scores with advancement from normal liver size to hepatomegaly ending with cirrhosis. Since the hippo pathway is a regulator of organ size, its inhibition causes activation of YAP and TAZ, causing hepatomegaly (39,40). Machado, et al. (33) proposed that transient YAP activation caused effective regeneration but scarring occurred if it persisted due to failure of inherent regulation of YAP expression.

Histologically, as portal and lobular inflammation represented a hallmark of chronic viral hepatitis, YAP and to a greater extent TAZ H scores showed significant association with increasing grades of portal inflammation, lymphoid aggregate formation, lobular confluent necrosis and necroinflammation grade of activity. This coincides with the results of Machado, et al. (33) study that showed significant association of YAP expression with portal inflammation and necro-inflammatory grade of non-alcoholic fatty liver disease (NAFLD) patients. Similarly, TAZ silencing in nonalcoholic steatohepatitis (NASH) patients caused a decrease in the inflammatory cell infiltrate together with cell death (41). The relation between Hippo pathway effectors and inflammation was clarified in a study postulating that full YAP activation required inflammatory stimuli through the cytokine receptor Glycoprotein 130 (gp130) and Tyrosine protein (Src) kinase beside the inhibitory LATS1/2 kinase pathway (42,43).

Owing to the fact that biliary ductular proliferation occurred as a reaction for fibrogenesis (44), the YAP and TAZ H score values showed correlation with ductular proliferation in the chronic viral hepatitis group that could also be observed in other studies (33,45,46).

YAP and TAZ could be activated via the mevalonate pathway which represents the main pathway for cholesterol biosynthesis. Moreover, statins are cholesterol lowering drugs and a mevalonate pathway blocker causes inactivation of YAP and TAZ leading to their cytoplasmic accumulation (47). This was reflected in our cases as both YAP and TAZ H scores showed significant correlation with steatosis in chronic viral cases but the H score appeared increased as we progressed from grade 1, to grade 2 then declined in grade 3. Verma, et al. (48) concluded that especially grade (1,2) steatosis was associated significantly with advanced fibrosis in HCV patients and thus we could expect more YAP and TAZ expression in those grades due to their relation with hepatic fibrosis, explaining their decreased mean values in grade 3.

Fibrosis ending with cirrhosis as an architectural distortion occurs as sequence of persistent parenchymal damage and chronic hepatic stellate cell activation. YAP and TAZ played a major role as early key transcriptional effectors in HSCs (19,49). YAP can mediate epithelial mesenchymal transition in hepatocytes as one of fibrosis mechanisms in the liver (20,50). Also, YAP overexpression in hepatocytes induced their dedifferentiation into hepatic progenitor stem cells ((HPC) oval cells) (32,33). HPC role in fibrosis was explained by two opinions: either stiff ECM activates HPC action or HPC promotes excess matrix deposition leading to cirrhosis (51). Because YAP and TAZ act as mechano-sensors for extracellular matrix stiffness, stiffening of ECM induces activation of YAP and TAZ in a continuous positive fibrotic feedback leading to cirrhosis (52,53). YAP expression appeared positively correlated with fibrosis stage in both studies of NAFLD (33) and neonatal cholestasis patients (46). This corresponded with the studied chronic viral hepatitis patients that showed progressive increase in both YAP and TAZ H scores with advancement of fibrosis stage making them independent prognostic factors of the H score in both YAP and TAZ linear regression analysis.

Clinically in the studied chronic viral hepatitis group, YAP alone showed direct clinical correlation with age, as with aging the hepatocyte wear and tear and regeneration increases (54) and also the fibrosis progresses with older age (55,56).

Regarding the laboratory results, a direct correlation appeared between YAP and TAZ H scores and liver enzymes. This occurred because hepatocyte injury resulted in release of both Hippo effectors and liver enzymes (33,57). Similarly, YAP was correlated with ALP in NAFLD patients in Machado, et al. (33) study and also TAZ silencing reduced ALT level in NASH (41). YAP showed an inverse correlation with serum albumin as albumin level deteriorated with progression of fibrosis (58).

YAP and TAZ H score values exhibited direct correlation in the studied chronic viral hepatitis cases which means that both act synergistically upon hepatocyte damage initiating fibrosis. Once the Hippo pathway becomes blocked, this causes the release of active YAP and TAZ with their nuclear translocation and transcriptional activation by the TEAD mediated complex leading to ECM deposition and ongoing fibrosis (20,21).

In the AIH group, owing to YAP’s role in autoimmunity, the H score values exhibited a direct correlation with serum ALT, AST and ALP levels as elevation of liver enzymes is an important feature of autoimmune hepatitis (28,37).

TAZ regulates the process of T cell differentiation via both RORγt and Foxp3 independent of TEADs transcription factors concerned with fibrosis. Adding to that, once TEAD1 becomes activated TAZ appeared sequestered away from RORγt ending with inhibition of TH17 proliferation (35). This prominent role of TAZ in initiating inflammation rather than fibrosis in autoimmune diseases could be proven by absence of statistical correlation between fibrosis stage and H score values of not only for TAZ but also for YAP in the studied AIH cases. Although, the H score values of both YAP and TAZ attained higher means as we progress from low to high grade of necroinflammation, this relation failed to reach statistical significance. The speculative role of YAP in autoimmune diseases needed to be clarified in further studies.

In addition to its crucial role in initiation and progression of fibrosis, YAP and TAZ activation was found to ameliorate the innate immunity against viruses. YAP/ TAZ antagonize the antiviral response through binding to TANK binding kinase 1 (TBK1) together with interferon regulatory factor 3 (IRF3) preventing the expression of type I interferon gene (59). Thus, YAP and TAZ downregulation could be promising in treating chronic viral hepatitis cases via regression of fibrosis stage together with activation of antiviral immune response. Several YAP and TAZ antagonists had been tried in research to induce reversal of fibrosis including fish oil (49), verteporfin (19), morin (60) and tetramethylpyrazine (61). Those drugs could be used in chronic viral hepatitis cases as alternative to liver transplantation with its massive cost.

Importantly, Machado et al. (33) found that silencing of TAZ in mouse models did not affect YAP expression. This means that blockage of one Hippo effector will not antagonize the other necessitating therapeutic double hit of both molecules.

Concerning its role in autoimmunity, knockdown of TAZ improved autoimmune diseases in mouse models (38). Also, digoxin antagonized RORγt receptor causing regression of autoimmune diseases (62). As both Hippo effectors act in a pathogenic manner completely different in AIH from that in chronic viral ones, YAP and TAZ could represent a very reasonable blocking target for a novel approach in treatment of autoimmunity.

This study provides a novel insight about the role of YAP and TAZ in development of fibrosis and scarring in chronic viral hepatitis patients that represents a major health problem in Egypt. It also points to a possibly different way of action of both Hippo effectors in AIH which need further research to delineate the specific mechanisms involved.

## Conflict of Interest

The authors declare no conflict of interest.
